# Polyamidoamine Dendrimers Decorated Multifunctional Polydopamine Nanoparticles for Targeted Chemo- and Photothermal Therapy of Liver Cancer Model

**DOI:** 10.3390/ijms22020738

**Published:** 2021-01-13

**Authors:** Bartosz F. Grześkowiak, Damian Maziukiewicz, Agata Kozłowska, Ahmet Kertmen, Emerson Coy, Radosław Mrówczyński

**Affiliations:** 1 NanoBioMedical Centre, Adam Mickiewicz University in Poznań, Wszechnicy Piastowskiej 3, PL-61614 Poznań, Poland; damian.maziukiewicz@amu.edu.pl (D.M.); agakoz@amu.edu.pl (A.K.); ahmker@amu.edu.pl (A.K.); coyeme@amu.edu.pl (E.C.); 2 Faculty of Physics, Adam Mickiewicz University in Poznań, Uniwersytetu Poznańskiego 2, PL-61614 Poznań, Poland; 3 Faculty of Chemistry, Adam Mickiewicz University in Poznań, Uniwersytetu Poznańskiego 8, PL-61614 Poznań, Poland

**Keywords:** polydopamine, dendrimers, PAMAM, combined therapy, cancer therapy, photothermal therapy

## Abstract

The development of multifunctional drug delivery systems combining two or more nanoparticle-mediated therapies for efficient cancer treatment is highly desired. To face this challenge, a photothermally active polydopamine (PDA) nanoparticle-based platform was designed for the loading of chemotherapeutic drug and targeting of cancer cells. PDA spheres were first functionalized with polyamidoamine (PAMAM) dendrimers followed by the conjugation with polyethylene glycol (PEG) moieties and folic acid (FA) targeting ligand. The anticancer drug doxorubicin (DOX) was then absorbed on the particle surface. We performed the physico-chemical characterization of this versatile material and we assessed further its possible application in chemo- and photothermal therapy using liver cancer cell model. These nanoparticles exhibited high near-infrared photothermal conversion efficacy and allowed for loading of the drug, which upon release in specifically targeted cancer cells suppressed their growth. Using cell proliferation, membrane damage, apoptosis, and oxidative stress assays we demonstrated high performance of this nanosystem in cancer cell death induction, providing a novel promising approach for cancer therapy.

## 1. Introduction

Cancer has become one of the world’s most devastating diseases, with an estimated 18.1 million new cancer cases and 9.6 million cancer deaths in 2018 [1]. For many decades, cancer treatment has been limited to only a few strategies, such as surgery, radiation therapy and chemotherapy. However, these therapeutic approaches are associated with some drawbacks including the high possibility of recurrence, limited therapeutic efficacy, and undesirable side effects. Thus, development of novel anticancer agents is necessary to improve the therapeutic effectiveness of cancer treatments.

Nanomedicine is a rapidly evolving area of science connecting nanotechnology with the biomedicine and pharmacy [2]. The arrival of nanoparticle (NPs) technology to the field has enabled the development of a wide range of novel therapeutic and diagnostic platforms for cancer treatment including drug/gene delivery, photothermal therapy, photodynamic therapy, magnetic therapy, radiotherapy and imaging [3]. The incorporation of NPs into drug formulation systems can positively affect pharmacokinetics, efficacy, safety, and targeting. Many nanopharmaceuticals have entered clinical practice, and plenty of them are being tested in clinical trials [2]. On the other hand, nanodrugs also have to cope with the issues concerning superior characterization, possible toxicity, a lack of specific regulatory guidelines, cost-benefit considerations, and vanishing enthusiasm among medical doctors [4]. Most NPs are recognized and eliminated as a foreign substance by immune system. The literature on nanoparticle-based drug carriers revealed that only 0.7% (median) of the administered nanoparticle dose was delivered to a solid tumor [5]. Therefore, an efficient tumor-specific drug delivery system (DDS) needs to be designed to provide prolonged circulation in the body with specific targeting towards tumors.

Nanoparticles would preferentially accumulate at the tumor site via passive and active targeting mechanisms. According to central paradigm of cancer nanomedicine, NPs penetrate through leaky vessels into tumor environment and use the unique intra-organ pressures of the tumors to be kept inside. This process is called enhanced permeability and retention effect (EPR) [6]. However, passive extravasation accounts only for 3–25% of the nanoparticles tumor accumulation [7]. According to recent studies, NPs can bind to endothelial cells and enter through them through active trans endothelial pathways involving formation of fenestrae and uptake in vesicles and cytoplasmic transport. Further development of nanoparticle technology enabled introduction of actively targeted ligands onto their surface, which recognize biological structures in tumors and exploit very specific binding interactions to further improve delivery [6]. Nanoparticle surface modification with (poly)saccharides, peptides, aptamers, monoclonal antibodies and small molecules [8] which bind to the overexpressed receptors on the cancer cells may increase cell-specific uptake via receptor mediated endocytosis.

The heterogeneous nature of cancer is manifested in relevant issues that interfere with the eradication of cancer cells, which include multidrug resistance, drug efflux capacity, narrow therapeutic window, and undesired side effects [9]. Thus, a deep understanding of these complicated phenomena is crucial to design precise and efficient therapeutic approach. Numerous studies promote the concept of a combination of two or more nanoparticle-based therapies resulting in a synergistic therapeutic outcome to improve common cancer treatments. To further increase survival rate of cancer patients, combinatorial strategies have been developed for delivering chemotherapeutics together with an energy-mediated approaches including photodynamic, hyperthermia, or radiotherapy [10].

Hyperthermia, a type of cancer treatment based on exposing body tissue within the 41–48 °C temperature range [11], contributes to alteration of tumor microenvironment [12], immune response [13], vascularization [14], and reduction of cell resistance against radiation [15,16]. The safe therapeutic window for this thermal-based treatment is between 41 and 45 °C. In this temperature range, the rate of biochemical reactions is significantly increased which lead to appearance of oxidative stress and the induction of apoptosis in the cells [17]. The tumor cells tend to become more sensitive to chemotherapy and radiation therapy when combined with hyperthermia, moreover, above 46 °C, rapid necrotic cell death is promoted [18]. In combinational therapies, hyperthermia can promote chemotherapeutic drug uptake by cancer cells (below 46 °C) or kill the cells due to the elevated temperature (above 46 °C). However, the temperature must be carefully controlled to avoid damage to adjacent healthy tissues. This can be achieved by incorporation of heating nanoparticles (HNPs) into cancer cells enabling high heat deposition in the tumor area and thus diminish the damage to the surrounding tissues [19].

The most well-known HNPs are those in which heating is induced by the application of oscillating magnetic fields [20,21]. An interesting alternative of magnetically induced hyperthermia offer light-activated HNP capable of efficient heat generation under irradiation by a light source with adequate wavelength [11]. They can be classified into different families such as metallic NPs, carbon-based NPs, semiconductor or rare earth ions doped nanocrystals, and organic nanoparticles [22]. Polydopamine (PDA), a mussel-inspired polymer [23], has found a wide application in photothermal therapy as member of organic nanoparticles class [24,25,26]. This is attributed to a high near-infrared (NIR) photothermal conversion efficiency of this polymer [27]. Moreover, PDA is characterized also by high biocompatibility [28] and ease of functionalization that allows for surface modification with PEG and tumor-targeting ligands [29]. It was demonstrated that PDA could be used as a nanocarrier for drug delivery. Drug can be encapsulated in a crosslinked structure of polydopamine [30], efficiently adsorbed on its surface [31] or entrapped in the frame of chemical compounds attached to the PDA [32]. In our previous research, the PDA-coated magnetic nanoparticles functionalized with mono-6-thio-β-cyclodextrin [33] or PAMAM dendrimers [32] were obtained and successfully applied in combined chemo- and photothermal therapy towards liver cancer cells in vitro. Nevertheless, the lack of highly efficient active targeting essential for the modern multifunctional nanostructures hinders their application in cancer treatment.

**Figure 1:**
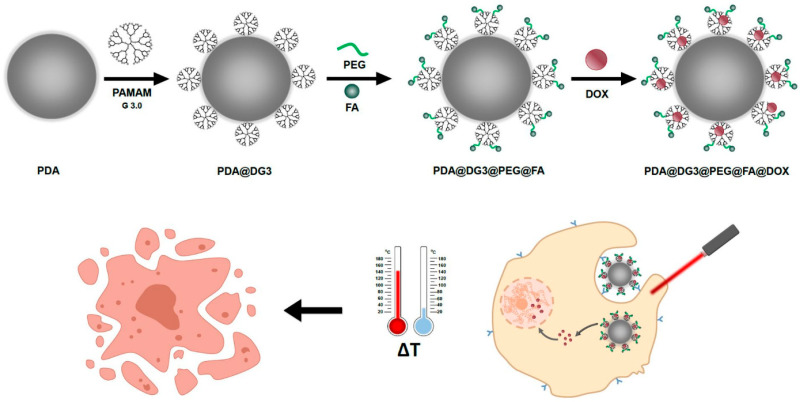
Schematic illustration of polydopamine (PDA)@PAMAM dendrimers G 3.0 (DG3)@polyethylene glycol (PEG) @folic acid (FA)@doxorubicin (DOX) NPs for targeted dual chemo and photothermal synergistic cancer therapy.

In this report, we present synthesis of a hybrid drug nanocarrier based on biomimetic polydopamine nanoparticles decorated with commercially available PAMAM dendrimers generation 3.0. We also demonstrate a universal strategy for peripheral amino groups functionalization using bifunctional linker via thiol-ene click chemistry that allows further modification of nanoparticles with folic acid (FA) for active targeting of cancer cells. Moreover, the capability of hydrophobic doxorubicin (DOX) encapsulation within the dendrimer frame along with strong photothermal properties of PDA core allow application of these NPs in combined chemo- and photothermal treatment of hepatocellular carcinoma (Figure 1). We discuss the mechanism of cell death induced by NIR laser irradiation combined with drug delivery using evaluation of reactive oxygen species (ROS) generation in the cells and apoptosis profile analysis. These results are of great interest in the area of hybrid materials development and their application in the field of nanomedicine for anticancer therapy.

## 2. Results and Discussion

### 2.1. Synthesis and Characterization of Materials

**Figure 2:**
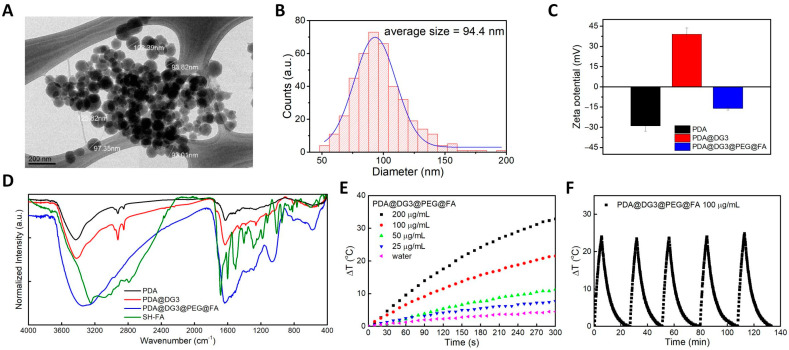
(**A**) Transmission electron microscopy (TEM) image and (**B**) size distribution of PDA@DG3@PEG@FA nanoparticles. (**C**) Zeta potential values (*n*-value = 3) and (**D**) Fourier transform infrared (FT-IR) spectra for PDA, PDA@DG3 and PDA@DG3@PEG@FA NPs. (**E**) Temperature curves of the PDA@DG3@PEG@FA solution with different concentrations under continuous NIR laser irradiation (808 nm, 2 W/cm^2^) for 5 min. (**F**) Temperature curve of the PDA@DG3@PEG@FA solution (100 µg/mL) under five NIR laser on/off cycles (808 nm, 2 W/cm^2^).

The schematic presentation of the synthesis process is presented in Figure S1. In this work, polydopamine spheres were obtained by oxidative polymerization of dopamine in the presence of sodium hydroxide at 50 °C. The size of the nanoparticles could be easily controlled by the amount of base added to the reaction [34]. The PDA cores as a part of a final nanocarriers were spherical in shape with relatively good size distribution according to TEM analysis (Figure 2A). As shown in the graph, the average size of the cores was about 94 nm (Figure 2B). DLS data show presence of populations of two differently sized PDA spheres (Figure S2). However, smaller ones constitute only small percentage of the entire sample. The zeta potential was also highly negative (Figure 2C) congruent with PDA NPs [35]. In the next step, PAMAM dendrimers generation 3.0 (G 3.0) were attached to PDA nanoparticles by Michael reaction between peripheral amino groups from PAMAM and quinone groups present in the PDA structure. After functionalization with PAMAM G 3.0, we observed zeta potential shift from negative (−29 ± 4.7 mV) to positive (39 ± 4 mV), what proves a successful decoration of PDA spheres with dendrimers. This value is an indicator of the high colloidal stability of these nanoparticles. The number of generations affects the size of the PAMAM polymer. Hydrodynamic diameter of G 3.0 was calculated to be 3.6 nm [36]. Surprisingly, no evident change in size was noticed in the DLS results (Figure S2). This is especially interesting as there were no other peaks in Zeta potential graph, which could suggest the presence of bare PDA spheres or free dendrimers. Then, the PAMAM G 3.0 coated PDA nanoparticles (PDA@DG3) were transferred to a one-pot reaction with bifunctional PEG linker ended with NHS and maleimide moieties (NHS-PEG-Mal) followed by addition of thiol-derivative of folic acid (SH-FA). First step of the reaction was carried out in the slightly basic pH to favor the interaction between free amine (−NH_2_) groups from PAMAM and NHS group from the linker and avoid side reaction between −NH_2_ moieties and maleimide. Next, pH was changed to allow thiol-ene click reaction between SH-FA. After functionalization of NPs with folic acid, the zeta potential value decreased to −16 ± 1.2 mV due to the presence of free carboxylic groups from FA, whereas the hydrodynamic diameter is increased to about 150 nm according to DLS measurements (Figure S2). It has been reported that this is the desired size in order to avoid the activation of the lymphatic system or very fast excretion from the circulation [37]. Fourier transform infrared (FT-IR) measurement of PDA@DG3@PEG@FA showed that the broad band between 3600 to 3300 cm^−1^ with main peak at 3431 cm^−1^ in PDA FTIR spectrum was assigned to stretching modes of ν(N−H), ν(O−H) from amino and hydroxyl moieties present in the PDA structure. The peaks at 2969, 2924 and 2851 cm^−1^ corresponded to stretching C-C modes from aliphatic CH_2_ groups presented in the mere PDA particles. The peak at 1730 cm^−1^ is due to the presence of carbonyl ν(C=O) from quinons. The signals between 1645 and 1340 cm^−1^ range resulted from stretching modes of ν ring (C=C), ν ring (C=N), from aromatic amines and indol ring, respectively. After functionalization with PAMAM dendrimers the peaks between 2969 cm^−1^ to 2850 cm^−1^ region became more intense due to the higher amount of CH_2_ units from PAMAM dendrimers. Moreover, these dendrimers usually show three characteristic bands at 3352 cm^−1^, 1596 cm^−1^ and 1453 cm^−1^ that can be assigned to NH stretch (primary amines), NH bending of N-substituted amide and CH bending, respectively [38]. However, in our case the peaks at 3352 cm^−1^ and 1596 cm^−1^ are superimposed with bands from polydopamine. We observed peak at 1457 cm^−1^ only which indicated attachment of PAMAM dendrimers to the PDA particles in the sample. After surface functionalization with bifunctional linker and attachment of thiol derivative of folic acid we observed broadening of the band in 3650–3000 cm^−1^ range. Due to many functional groups presented in our nanoplatform that give signal in a similar range the unambiguous assignment is not possible. However, we could easily distinguished a strong and relatively broad band at 1065 cm^−1^ that resulted from PEG chain and was assigned to stretching vibration of C-O-C.

The photothermal properties of PDA@DG3@PEG@FA NPs were investigated in order to check if they could be used as efficient light-activated heating nanoparticles for photothermal therapy. For this purpose, aqueous dispersions of NPs were irradiated with a laser beam of 808 nm at power density of 2 W/cm^2^ for 300 s. As shown in the Figure 2E, PDA@DG3@PEG@FA NPs exhibited concentration- and time-dependent temperature increase upon NIR laser irradiation. The temperature was elevated by 33 °C at the highest concentration (200 µg/mL), whereas the temperature of pure water was slightly increased under the same conditions. It demonstrates that the photothermal effects arise from PDA NPs characterized by relatively high absorption coefficient (7.3 × 10^8^ M^−1^ cm^−1^) and photothermal efficiency of about 40% at 808 nm [27]. Finally, we evaluated the photostability of PDA@DG3@PEG@FA NPs by performing four cycles of ‘‘laser-on’’ and ‘‘laser-off’’ experiments. It could be seen that negligible decrease in temperature variations were recorded over five consecutive irradiation cycles (Figure 2F), indicating that PDA@Dg.3@PEG@FA maintained stable photothermal properties. All these results confirmed that analyzed NPs could be promising agents effectively absorbing and converting NIR light into heat.

In the proposed PDA@DG3@PEG@FA platform, the encapsulation of small-molecule drugs can occur in several ways, for example, the structure of the dendrimers allows to covalently attach drug molecules as well as incorporate them within the dendrimer cavities. The nature of the encapsulation involves electrostatic interactions, simple physical entrapment, and hydrophobic interaction and hydrogen bonding [39]. On the other hand, the PDA surface could be used for effective drug adsorption, in particular DOX, through π-π stacking and pH responsive controlled drug release [31]. Drug loading capacity was calculated to be 33% and 42% for PDA@DG3 and PDA@DG3@PEG@FA, respectively. The higher amount of drug loading for PDA@DG3@PEG@FA NPs may result from better interaction between drug and surface terminal groups conjugated with folic acid [40] as well as noncovalent interactions. As indicated by Golshan et al., hydrogen bonding and hydrophobic interaction between DOX and poly(propylene imine) (PPI) dendrimers could lead to physical entrapment of drug molecules inside the core and branches of dendrimer [41].

### 2.2. Photothermal Therapy of Liver Cancer Cells

It is well known that the toxicity of NPs depends on many factors including their size, shape, chemical composition and surface properties [42]. In this work, the cytotoxic effect of NPs at every stage of the synthesis process was evaluated using colorimetric WST-1 and fluorescent Live/Dead assays in normal liver epithelial cells (Figure S3). The results revealed concentration dependent toxicity according to cell metabolic activity measurement (Figure S3A). On the contrary, in Live/Dead cell viability assay, the ratios of viable to dead cells after incubation with tested NPs were alike to control cells (Figure S3B). We noticed that the cells remain vital, however their proliferation activity was hampered with increasing concentration of particles. At higher concentrations the type of particle functionalization affected the cell viability. The best survival profile was obtained for pure PDA spheres. These results are in agreement with the recent reports revealing that PDA covered nanomaterials show a reduced toxicity [28]. It was shown that the coating of PDA spheres with PAMAM dendrimers decreased the cell viability (Figure S3A,B). This may be the result of the positive charge of the NPs and enhanced interaction with negatively charged membranes [43]. Functionalization of PDA@DG3 NPs with PEG and FA further reduced cell metabolic activity at the highest concentration (40 µg/mL). As shown in the images in Figure S3C, despite the high live to dead cells ratio, we observed also a smaller number of cells at this concentration, as compared to pure PDA spheres and particles functionalized with PAMAM.

**Figure 3:**
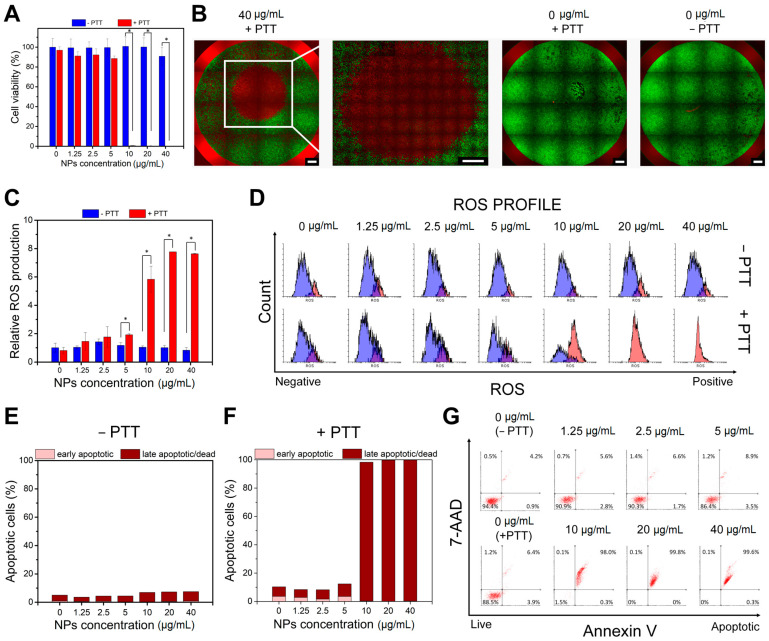
Cell viability assays of HepG2 cells incubated with different concentrations of PDA@DG3@PEG@FA NPs w/o (−PTT) and after laser irradiation (+PTT) with a power density of 2 W/cm^2^ for 5 min. (**A**) WST-1 cell viability assay results. (**B**) Fluorescence microscopy images; Calcein-AM stains for live cell (green); PI stains for dead cells (red); Scale bar represents 1000 μm. (**C**) Relative ROS production and (**D**) ROS profiles evaluated by flow cytometry (blue—ROS negative cells; red—ROS positive cells). (**E**,**F**) The percentages of cells in different phases and (**G**) apoptosis profile evaluated by flow cytometry. Statistically significant differences were indicated with an asterisk (*) for *p* < 0.05.

In the next step, in vitro PTT effect of PDA@DG3@PEG@FA NPs on hepatocellular carcinoma HepG2 cells was evaluated by WST-1 assay, together with Live/Dead cell staining, ROS generation and apoptosis profile determination. As can be seen in Figure 3A, cells maintained high survival rate in the absence of laser irradiation. By contrast, significantly reduced viability after laser (2 W/cm^2^) irradiation for 5 min for cells treated with increased concentration of NPs was observed. NPs presented a complete cell death at a concentration of 10 µg/mL, 20 µg/mL and 40 µg/mL in laser treated group (+PTT) after 48 h of incubation. These results demonstrated the excellent photothermal therapy ability of analyzed materials. Calcein-AM and ethidium homodimer-1 (EthD-1) labeling combined with fluorescence microscopy imaging was applied to further confirm the laser irradiation effect on tumor cells incubated with the PDA@DG3@PEG@FA NPs. As shown in Figure 3B, NPs treated cells exhibited red fluorescence signal at the highest concentration (40 µg/mL). It indicates membrane disruption during irradiation process because EthD-1 is a cell impermeable dye that can only stain dead cells. These data are consistent with the WST-1 assay results. A negligible reduction in cell viability was observed for the control group without any treatment (0 µg/mL −PTT) as well as the control group with laser irradiation (0 µg/mL +PTT). Additionally, quantitative analysis of cells undergoing oxidative stress after PTT was performed by flow cytometry (Figure 3C,D). It is considered that ROS generation is one of the crucial factors associated with apoptotic process [44]. The ROS level was measured in HepG2 cells incubated for 4 h with PDA@DG3@PEG@FA NPs immediately (0 h), 24 h or 48 h after laser treatment. As presented in Figure 3C,D, the relative ROS production remains unchanged with the increasing concentration of NPs. However, a significant increase in intracellular ROS level was observed right after the irradiation with the laser beam at concentrations above 5 µg/mL. Moreover, elevated ROS level was still observed for 24 h (Figure S4A) and 48 h (Figure S4B) after NIR laser irradiation. These data revealed that oxidative stress was involved in the cytotoxic process induced by both nanoparticles and NIR laser irradiation [45]. Treatment of cells only with laser beam did not trigger increment in ROS level. Particles themselves also did not cause such high increase of intracellular ROS. On the other hand, Mocan et al. showed that hyperthermia caused by PEG-ylated multi-walled carbon nanotubes (MWCNTs-PEG) laser mediated treatment contributed to mitochondrial membrane depolarization [46]. This process activated the flux of free radicals into cell and the oxidative state induced apoptotic-mediated cellular damage in pancreatic cancer cells. Apoptosis profile analysis of cells incubated with PDA@DG3@PEG@FA NPs and undergoing NIR laser irradiation was performed using flow cytometry. As shown in Figure 3E, we did not observe a significant increase in the rate of apoptotic cells after incubation with analyzed NPs. However, after laser irradiation the late apoptotic/dead cell population reached as high as 100% at high nanoparticles concentration (10–40 µg/mL) (Figure 3F,G). This indicates that near-total cell damage was induced by nanoparticles via photothermal effect. At temperatures higher than 50 °C primarily necrosis is observed, whereas below ~50 °C primarily cell apoptosis is caused [47]. According to the result in Figure 2E, the increment of the temperature do not exceed 50 °C after laser irradiation. We hypothesize that apoptosis was the primary mechanism responsible for HepG2 cell death triggered by PTT at higher temperatures. However, we cannot exclude that necroptosis is also involved in the cytotoxic process since it can be induced by the laser irradiation. It has been reported that laser treatment of gold nanorods with a targeting adaptor FA (GNR-FA) caused cells death and necroptosis regulated by RIPK1 pathway plays a key role in the killing process of melanoma tumor cells [47]. Based on the in vitro PTT experiments, we can claim that the analyzed nanoparticles were capable of efficient damaging of tumor cells through photothermal ablation and their toxicity was low without laser irradiation.

### 2.3. Chemotherapy of Liver Cancer Cells

**Figure 4:**
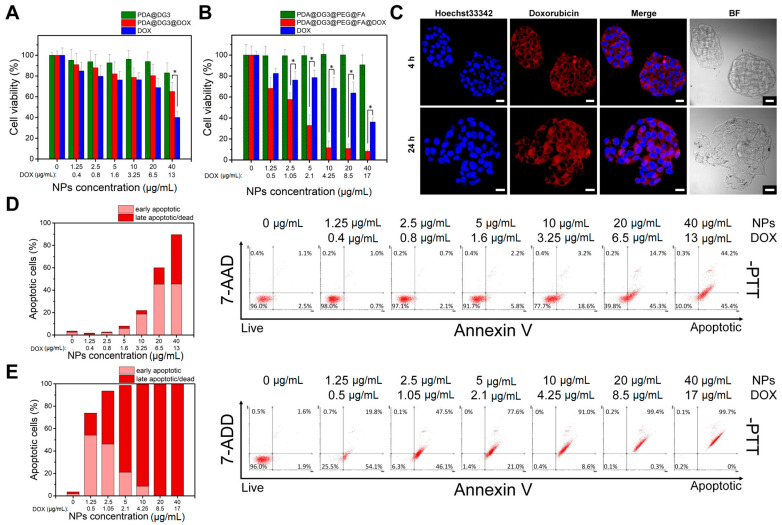
Cell viability assays of HepG2 cells incubated with different concentrations of DOX loaded PDA@DG3 and PDA@DG3@PEG@FA NPs. (**A**,**B**) WST-1 cell viability assay results. (**C**) Confocal laser scanning microscopy images of cells incubated with PDA@DG3@PEG@FA@DOX NPs for 24 h; Hoechst 33342 stained nuclei (blue); DOX (red); Scale bar represents 20 μm. The percentages of cells in different phases and apoptosis profile evaluated by flow cytometry of (**D**) PDA@DG3@DOX and (**E**) PDA@DG3@PEG@FA@DOX. Statistically significant differences were indicated with an asterisk (*) for *p* < 0.05.

Due to unique properties such as remarkable adhesiveness, excellent biocompatibility, simple synthesis requirements and different drug loading approaches, PDA-based NPs have been widely used as drug carriers [48]. In this work, PDA@DG3 and PDA@DG3@PEG@FA NPs loaded with DOX were incubated with HepG2 cells and WST-1 assay was performed to investigate their chemotherapeutic effect. As shown in Figure 4A, a DOX concentration dependent decrease in cell viability was observed for drug-loaded NPs. However, we did not observe any advantage over the treatment of cells with pure DOX. In contrast to PDA@DG3@DOX NPs, those functionalized with folic acid had significantly reduced effect on cell viability as compared to free doxorubicin (Figure 4B). At the highest concentrations the survival rate dropped below 20%. These data suggest that PDA@DG3@PEG@FA NPs could deliver drug with enhanced efficiency due to the active targeting of folate receptors (FR) on cell membranes. The FR is a recognized biomarker for cancer cells due to its overexpression on multiple tumors including liver cancer [49]. To evaluate cellular uptake of the particles confocal laser scanning microscopy was performed. As shown in Figure 4C, high uptake efficiency of PDA@DG3@PEG@FA@DOX nanoparticles after 4 and 24 h of incubation with HepG2 cells was observed. High internalization of doxorubicin results from the enhanced interaction of folic acid-conjugated NPs with the cellular membrane. To further analyze the cell death mechanism caused by drug loaded NPs, apoptosis profile in HepG2 cells was analyzed using flow cytometry. Figure 4D revealed, that after 48 h of exposure, the early apoptotic rate was raised with increasing NPs concentration in PDA@DG3@DOX treated group. While the late apoptotic/dead cells rate was elevated at 40 µg/mL. On the other hand, the early apoptotic cells rate was much lower than the late apoptotic/dead cells rate induced by PDA@DG3@PEG@FA@DOX NPs (Figure 4E). These data demonstrated that for FA-functionalized NPs apoptotic mechanism is responsible for cell death and therapeutic effect can be achieved at very low concentration of drug. We can conclude that doxorubicin could be delivered with high efficiency and that the chemotherapy treatment is thus more effective, what is in consistence with WST-1 cell viability assay results.

### 2.4. The Activity of Nanomaterials in Combined Chemo- and Photothermal Liver Cancer Therapy

**Figure 5:**
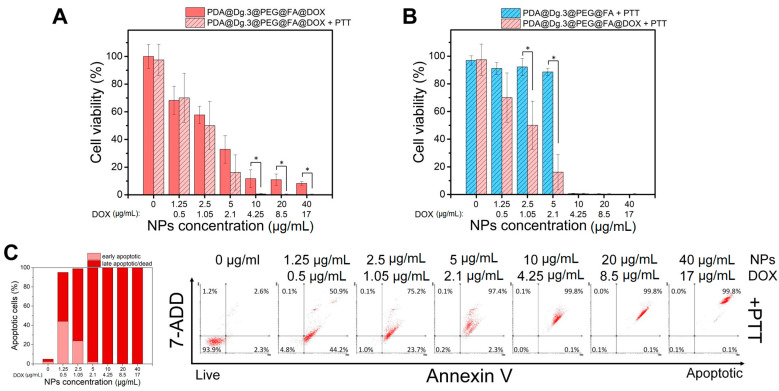
Cell viability assays of HepG2 cells incubated with different concentrations of DOX loaded PDA@DG3@PEG@FA NPs after laser irradiation with a power density of 2 W/cm^2^ for 5 min. (**A**,**B**) WST-1 cell viability assay results. (**C**) The percentages of cells in different phases and apoptosis profile evaluated by flow cytometry. Statistically significant differences were indicated with an asterisk (*) for *p* < 0.05.

In the further stage, we investigated the possible application of PDA@DG3@PEG@FA@DOX NPs in combined chemo- and photothermal therapy (CT-PTT). For this purpose, the HepG2 cells were irradiated with NIR laser after 4 h incubation with the NPs and the cell viability assays were performed after additional 48 h. We did not observe the advantage over the treatment of cells with NIR laser at lower concentrations (1.25–2.5 µg/mL) as compared to standard chemotherapy (Figure 5A). However, in the range of concentrations between 5–40 µg/mL, a drop in cell viability after laser irradiation was demonstrated. This phenomenon was attributed to the increasing concentration of PDA containing NPs that enhanced the photothermal effect and resulted in a more effective eradication of cancer cells in comparison to chemotherapeutic approach. As shown in Figure 5B, decreased cell survival in the concentration range between 1.25 and 5 µg/mL was connected with application of drug-loaded NPs. It was found that dual therapy contributes to lower tumor growth and cancer regression due to more efficient drug penetration to the associated cells in the tumor tissue [50,51]. Despite negligible effect of cell viability decrease in +PTT treated group in terms of metabolic activity, the late apoptotic/dead cells rate was elevated in the concentration range between 1.25 and 5 µg/mL (Figure 5C) as compared to non-irradiated cells (−PTT) (Figure 4E). It may result from enhanced drug release from the particles at lower concentrations due to application of NIR laser irradiation [52,53,54].

## 3. Materials and Methods

### 3.1. Materials

Dopamine hydrochloride was purchased from Alfa Aesar (Gdansk, Poland). Doxorubicin hydrochloride (DOX·HCl) was purchased from LC Laboratories (Boston, MA, USA). PAMAM dendrimers G 3.0, phosphate buffer saline (PBS), Menadione, sodium tetraphenylborate, tris (hydroxymethyl) aminomethane, *O*-[*N*-(3-Maleimidopropionyl)aminoethyl]-*O*′-[3-(*N*-succinimidyloxy)-3-oxopropyl]heptacosaethylene glycol (molecular weight of 1570.76 g/mol), fetal bovine serum (FBS), penicillin, streptomycin, fibronectin, bovine collagen type I, bovine serum albumin, 0.1% poly-l-lysine, Muse^®^ Oxidative Stress Assay and MUSE^®^ Annexin V and Dead Cell assay were purchased from (Merck Darmstadt, Germany). Bronchial Epithelial Basal Medium (BEBM) and BEGM Bullet Kit were purchased from Lonza (Basel, Switzerland). WST-1 Cell Proliferation Reagent was purchased from Takara Bio (Shiga, Japan). Calcein AM, ethidium homodimer-1, Hoechst 33342, Minimum Essential Medium Eagle (MEM), dulbecco’s phosphate buffered saline (DPBS), 4% formaldehyde solution, sodium pyruvate, sodium pyruvate were purchased from Thermo Fisher Scientific (Waltham, MA, USA).

### 3.2. Synthesis of PDA@DG3@PEG@FA NPs

Pure PDA spheres were synthesized according to previously reported protocol with slight modification [34]. Briefly, dopamine hydrochloride (100 mg, 10 mM) was dissolved in 200 mL of Milli-Q water and 1 mL of 1 M NaOH was added when the temperature of the solution reached 50 °C. Reaction was continued for 2 h followed by washing of the NPs with EtOH and Milli-Q water. Next, 6.75 mg of PDA particles were dispersed in 6 mL of TRIS buffer (pH 8.5, 10 mM) and 300 μL of PAMAM dendrimers generation 3.0 suspended in MeOH was added. After 4 h of stirring, particles were centrifuged for 10 min at 20,000 rpm at RT and washed with Milli-Q water. Immediately after the dendrimer attachment, PAMAM functionalized PDA particles were dispersed in 6 mL of borate (NaB) buffer (pH 8.5; 10 mM). Then, Mal-PEG-NHS linker was added to PDA@DG3 particle dispersion at the Mal-PEG-NHS to PAMAM wt/wt ratio of 4:1. Reaction was carried out for 1.5 h at RT followed by NaB buffer replacement with phosphate buffered saline (PBS) buffer (pH 6.8). Next, 16 mg of thiol-derivative of folic acid (SH-FA) obtained according to the literature protocol [55] was added to the suspension of PDA@DG3@PEG-Mal NPs and reaction was continued o/n in the dark. Finally, particles were collected by centrifugation and washed with Milli-Q water.

### 3.3. Characterization of PDA@DG3@PEG@FA NPs

Transmission electron microscopy (TEM) micrographs were recorded on a JEM-1400 microscope (JEOL, Tokyo, Japan) working at an accelerating voltage of 120 kV. Samples were drop casted on a copper grid (Formvar/Carbon, TedPella, Redding, CA, USA). Fourier transform infrared (FT-IR) spectra were recorded on Vertex 70 spectrometer (Bruker, Germany) in KBr pellets. Zeta potentials and hydrodynamic diameters were measured using Zetasizer Nano ZS (Malvern Instruments Ltd., Malvern, United Kingdom). Photothermal and photostability experiments were performed at 808 nm wavelength NIR laser at a power density of 2 W/cm^2^ (Changchun New Industries Optoelectronics Tech. Co. Ltd., Changchun, People’s Republic of China). The temperature measurements were collected with a 10 s interval over a 5 min period.

### 3.4. Drug Loading

PDA@DG3 or PDA@DG3@PEG@FA NPs (2 mg) were mixed with 2 mL of doxorubicin (1 mg/mL) in PBS buffer (10 mM, pH 7.4) and the mixtures were shaken at 23 °C for 24 h. Then, the nanocarriers were collected by centrifugation and washed two times with 2 mL of PBS buffer. The amount of DOX content was obtained from the drug concentrations before and after loading by means of UV- vis absorption at 486 nm (Perkin-Elmer Lambda 950 UV/Vis/NIR, Waltham, MA, USA). 1 mg of nanoparticles per 1 mg of DOX was used, therefore encapsulation efficiency (EE) value corresponded to loading capacity (LC) value.

### 3.5. Cell Culture

HepG2 hepatocellular carcinoma cell line and THLE-2 epithelial cell line isolated from the human liver were purchased from American Type Culture Collection (ATCC, Manassas, VA, USA) and maintained at 37 °C in a 5% CO_2_ humidified environment. HepG2 cells were cultured in a Minimum Essential Medium Eagle (MEM) medium supplemented with 10% Fetal Bovine Serum (FBS), 1% antibiotics (penicillin 100 µg/mL, streptomycin 100 µg/mL), non-essential amino acids and sodium pyruvate. THLE-2 cells were cultured in a BEBM medium supplemented with BEGM Bullet Kit, 10% FBS and 1% antibiotics. The flasks and plates for the THLE-2 culturing were precoated with a coating solution of 0.01 mg/mL fibronectin, 0.03 mg/mL bovine collagen type I and 0.01 mg/mL bovine serum albumin dissolved in MEM medium.

### 3.6. Cell Viability Assays

HepG2 and THLE-2 cells were seeded at the density of 3×10^4^ and 1×10^4^ cells per well in the 96-well plate, respectively. After 24 h of cultivation, the increasing concentrations of tested nanoparticles (1.25–40 µg/mL) were added to each well, and the cells were further incubated for defined period of time. To verify the cell viability after laser treatment, HepG2 cells were incubated with increasing concentration of NPs (1.25–40 µg/mL) for 4 h. After this time, cells were irradiated by 808 nm laser with laser power of 2 W/cm^2^ for 5 min/well and further incubated for defined period of time. Cell viability was determined using metabolic activity, membrane integrity, oxidative stress and apoptosis assays. The non-treated cells (w/o nanoparticles) and the non-treated cells (w/o nanoparticles) irradiated with the laser were used as a negative controls.

#### 3.6.1. Metabolic Activity Assay

To evaluate viability of the cells incubated with nanoparticles, the WST-1 cell proliferation assay was carried out after 48 h. Briefly, 10 µL of the WST-1 Cell Proliferation Reagent was added to each well and the cells were incubated for 4 h. To avoid nanoparticle-mediated light absorption, 100 µL of supernatant was transferred to fresh wells. The absorbance was recorded at 450 nm (reference wavelength 620 nm) against the background control using a multi-well plate reader (Zenyth, Biochrom, Cambridge, UK). The cell viability was exhibited as the respiration activity normalized to untreated cells (w/o nanoparticles). All experiments were carried out in triplicates.

#### 3.6.2. Live/Dead Cell Assay

To perform Live/Dead cell viability assay, THLE-2 cells were seeded in black polystyrene 96-wells flat bottom plate with the transparent bottom (Greiner Bio-One GmbH, Kremsmünster, Austria) precoated with the coating solution. After 48 h incubation with the nanoparticles, cells were labeled with 2 µM calcein AM, 2 µM ethidium homodimer-1 and 8 µM Hoechst 33342 diluted in dulbecco’s phosphate buffered saline (DPBS) (100 µL/well) for 30 min at 37 °C. Subsequently, the cells were imaged using the IN Cell Analyzer 2000 (GE Healthcare Life Sciences, Pittsburgh, PA, USA). Living cells were imaged using the FITC/FITC excitation/emission filters while for the dead cells, the TexasRed/TexasRed ex/em filters were applied. DAPI/DAPI was used to image the Hoechst 33342 blue signal. Twenty fields of view were taken per well with a 20× magnification. Evaluation of the obtained images was conducted using the IN Cell Developer Toolbox software (GE Healthcare Life Sciences) using an in-house developed protocol. In a first step, the total cell number was calculated from the DAPI images. Finally, the number of dead cells from the TexasRed images was established.

To image live and dead cells, HepG2 cells seeded at the density of 1.2 × 10^5^ cells per well in 24-well plate coated with 0.01% poly-l-lysine were incubated with 40 µg/mL of PDA@DG3@PEG@FA NPs. After 4 h of incubation, the cells were irradiated by 808 nm laser with power of 2 W/cm^2^ for 5 min. Following 24 h incubation, the cells were stained with 2 µM calcein AM and 2 µM ethidium homodimer-1 containing DPBS (250 µL/well) for 30 min at 37 °C. Subsequently, the IN Cell Analyzer 2000 was used to image the cells.

#### 3.6.3. Oxidative Stress Assay

To quantitatively measure cellular population undergoing oxidative stress based on detection of ROS, namely superoxide radicals, the Muse^®^ Oxidative Stress Assay was performed. Briefly, after 4 h, 24 h and 48 h incubation of cells with NPs, the cells were trypsinized, centrifuged and resuspended in 1x Assay Buffer. Next, 10 µL of cell in suspension was mixed with 190 µL of the MUSE^®^ Oxidative Stress working solution followed by 30 min incubation at 37 °C in the dark. The stained cells were then analyzed using MUSE^®^ Cell Analyzer (Merck). Non-treated cells (w/o nanoparticles) and the cells treated with 200 µM Menadione were used as a negative and positive control, respectively.

#### 3.6.4. Apoptosis Assay

To quantitatively determine live, early and late apoptosis, and cell death the MUSE^®^ Annexin V and Dead Cell assay was performed. Briefly, after 48 h of incubation, the cells were trypsinized, centrifuged and resuspended in PBS buffer containing 1% FBS. Next, 100 µL of cell in suspension was mixed with 100 µL of the MUSE^®^ Annexin V and Dead Cell reagent followed by 20 min incubation at RT in the dark. The stained cells were then analyzed using MUSE^®^ Cell Analyzer. Non-treated cells (w/o nanoparticles) and the cells treated with 5 µg/mL DOX were used as a negative and positive control, respectively.

### 3.7. Cellular Uptake

To show uptake of drug-loaded NPs, HepG2 cells were plated at the density of 7.5 × 10^4^ cells/well onto an 8-well Nunc^®^ Lab-Tek^®^ Chamber Slide (Thermo Fischer Scientific) and cultured for 24 h. After this time, 50 µL of PDA@DG3@PEG@FA@DOX NPs (5 µg/mL) per well was added and cells were incubated for 4 and 24 h. Following the fixation with 4% formaldehyde in DPBS buffer for 15 min, the cell nuclei were labeled with Hoechst 33342 at a concentration of 8 µM. Cell imaging was performed using a confocal laser scanning microscope (Olympus FV1000, Tokyo, Japan). Image acquisition and analysis were conducted with a 60× objective, a 1.4 oil immersion lens and FV10-ASW software (Olympus). Doxorubicin fluorescence was imaged using 488 nm excitation and 560–590 nm emission filters, while Hoechst 33342 fluorescence was detected using 405 nm excitation and 425–472 nm emission filters.

### 3.8. Statistical Analysis

All quantitative data are represented as the mean ± standard deviation. Statistical analyses of cell viability results were performed using STATSOFT Statistica 10 software (StatSoft Power Solutions, Inc., Tulsa, OK, USA) and analysis of variance (ANOVA) (parametric or nonparametric) with subsequent post hoc analysis. Statistical significance was assumed for *p*-value < 0.05.

## 4. Conclusions

Based on the in vitro cell studies, we have shown that synthetized nanoparticles can be successfully used in combined chemo- and photothermal therapy of liver cancer. PDA NPs were efficiently functionalized with PAMAM G 3.0 dendrimers, PEG and folic acid. Nanoparticles exhibited sufficient properties to be used in photothermal therapy. They showed enhanced cytotoxicity in hepatocellular carcinoma cells after near-infrared laser irradiation at a laser power density of 2 W/cm^2^ according to colorimetric, Live/Dead, apoptosis and oxidative stress assays. Generation of ROS during laser irradiation is one of the mechanisms involved in cytotoxic process. Functionalization of NPs with PEG and FA improved chemotherapeutic effect as compared to dendrimer coated NPs. According to confocal fluorescence microscopy, analyzed nanoparticles delivered chemotherapeutic drug with high efficiency. Combined chemo- and photothermal therapy further decreased cell viability. Thus, our results are of great importance in the field of dendrimers modified nanoparticles and shed a new light on their characterization, functionalization and application in advanced anticancer therapy.

## Data Availability

The data presented in this study are contained within the article.

## Supplementary Materials

Supplementary materials can be found at https://www.mdpi.com/1422-0067/22/2/738/s1. Figure S1: Schematic presentation of synthesis procedure of PDA@DG3@PEG@FA NPs. Figure S2: Transmission electron microscopy images, dynamic light scattering and zeta potential measurements of PDA, PDA@DG3 and PDA@DG3@PEG@FA NPs. Figure S3: Cell viability assays results of THLE-2 cells incubated for 48 h with PDA, PDA@DG3 and PDA@DG3@PEG@FA NPs. Figure S4: Relative ROS production results and ROS profiles evaluated by flow cytometry for HepG2 cells incubated with PDA@DG3@PEG@FA NPs after 24 h and 48 h of irradiation with 808 nm laser (2 W/cm^2^, 5 min). Figure S5: Apoptosis profile of HepG2 cells incubated with PDA@DG3@PEG@FA NPs for 48 h evaluated by flow cytometry.

## Abbreviations

DDS	drug delivery system
DG3	dendrimers generation 3.0
DLS	dynamic light scattering
DOX	doxorubicin
EPR	enhanced permeability and retention effect
EthD-1	ethidium homodimer-1
FA	folic acid
FBS	fetal bovine serum
FT-IR	Fourier transform infrared
HNPs	heating nanoparticles
MEM	Minimum Essential Medium Eagle
NHS	N-Hydroxysuccinimide
NIR	near-infrared
PAMAM	polyamidoamine
PBS	phosphate buffered saline
PDA	polydopamine
PEG	polyethylene glycol
PPI	poly(propylene imine)
PTT	photothermal therapy
ROS	reactive oxygen species
SH-FA	thiol-derivative of folic acid
TEM	transmission electron microscopy
