# Supplementary material for: Polyamidoamine Dendrimers Decorated Multifunctional Polydopamine Nanoparticles for Targeted Chemo- and Photothermal Therapy of Liver Cancer Model
Source: Int J Mol Sci. 2021 Jan 13;22(2):738. doi: 10.3390/ijms22020738 (PMC7828497; doi:10.3390/ijms22020738)
Supplement: Supplementary file 1 [file ijms-22-00738-s001.pdf]

Supplementary

Polyamidoamine Dendrimers Decorated Multifunctional Polydopamine Nanoparticles for Targeted Chemo- and Photothermal Therapy of Liver Cancer Model

Bartosz F. Grześkowiak [1,*], Damian Maziukiewicz [1,2], Agata Kozłowska [1], Ahmet Kertmen [1,2], Emerson Coy [1] and Radosław Mrówczyński [1,3,*]

1 NanoBioMedical Centre, Adam Mickiewicz University in Poznań, Wszechnicy Piastowskiej 3, PL-61614 Poznań, Poland; damian.maziukiewicz@amu.edu.pl (D.M.); agakoz@amu.edu.pl (A.K.); ahmker@amu.edu.pl (A.K.); coyeme@amu.edu.pl (E.C.)
2 Faculty of Physics, Adam Mickiewicz University in Poznań, Uniwersytetu Poznańskiego 2, PL-61614 Poznań, Poland
3 Faculty of Chemistry, Adam Mickiewicz University in Poznań, Uniwersytetu Poznańskiego 8, PL-61614 Poznań, Poland
* Correspondence: bartoszg@amu.edu.pl (B.F.G.); radoslaw.mrowczynski@amu.edu.pl (R.M.)

Citation: Grze, B.F.; Maziukiewicz, D.; Koz, A.; Kertmen, A.; Coy, E.; Radosław M. Polyamidoamine Dendrimers Decorated Multifunctional Polydopamine Nanoparticles for Targeted Chemo- and Photothermal Therapy of Liver Cancer Model. *Int. J. Mol. Sci.* **2021**, 22, x. https://doi.org/10.3390/xxxxx

Received: 20 December 2020
Accepted: 11 January 2021
Published: date

Publisher's Note: MDPI stays neutral with regard to jurisdictional claims in published maps and institutional affiliations.

Copyright: © 2021 by the authors. Submitted for possible open access publication under the terms and conditions of the Creative Commons Attribution (CC BY) license (http://creativecommons.org/licenses/by/4.0/).

Supplementary Materials

Answer to the reviewer's comment regarding the issue of PEG and PAMAM ratio.

The wt/wt ratio between Mal-PEG-NHS and PAMAM dendrimers was 4:1 (more PEG chains). 17 times more PEG chains than PAMAM dendrimers (molar ratio) was used which is more than a half of available amino groups. If the reaction between PEG chains and PAMAM amino groups is 100 %, 17 out of 32 amino groups are covered. We assume that at least 25% (8) of amino groups are used for attachment to PDA. Therefore, 25 (17 from molar ratio and 8 used for PDA connection) out of 32 amino groups were occupied. It means that nearly 80% of amino groups are covered. If we omit those 8 groups used for attachment to PDA, then we have 17 out of 24 groups which equal to 70 %. Following the logic that one FA moiety covered one PEG chain, roughly 70 % of functional groups are covered with FA. We think that it is enough to change the charge of the particles from negative to positive.

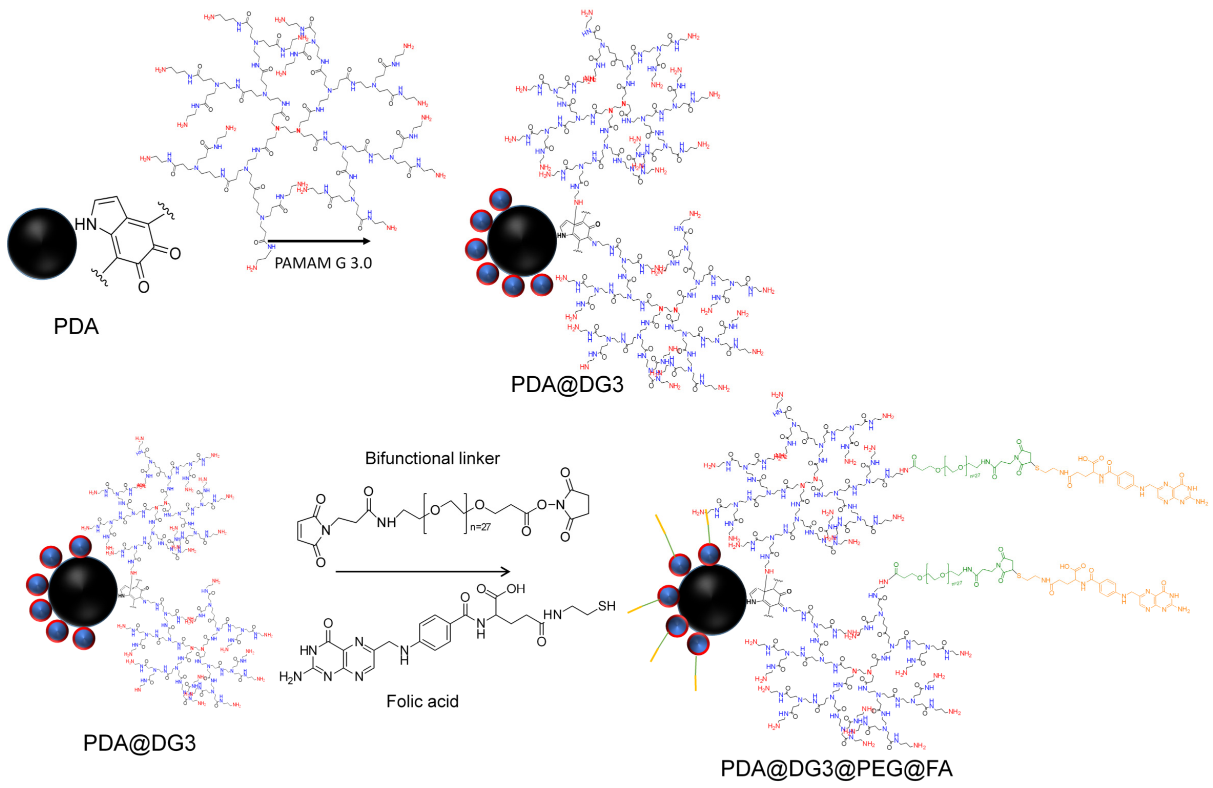

Figure S1. Schematic presentation of synthesis procedure of PDA@DG3@PEG@FA NPs.

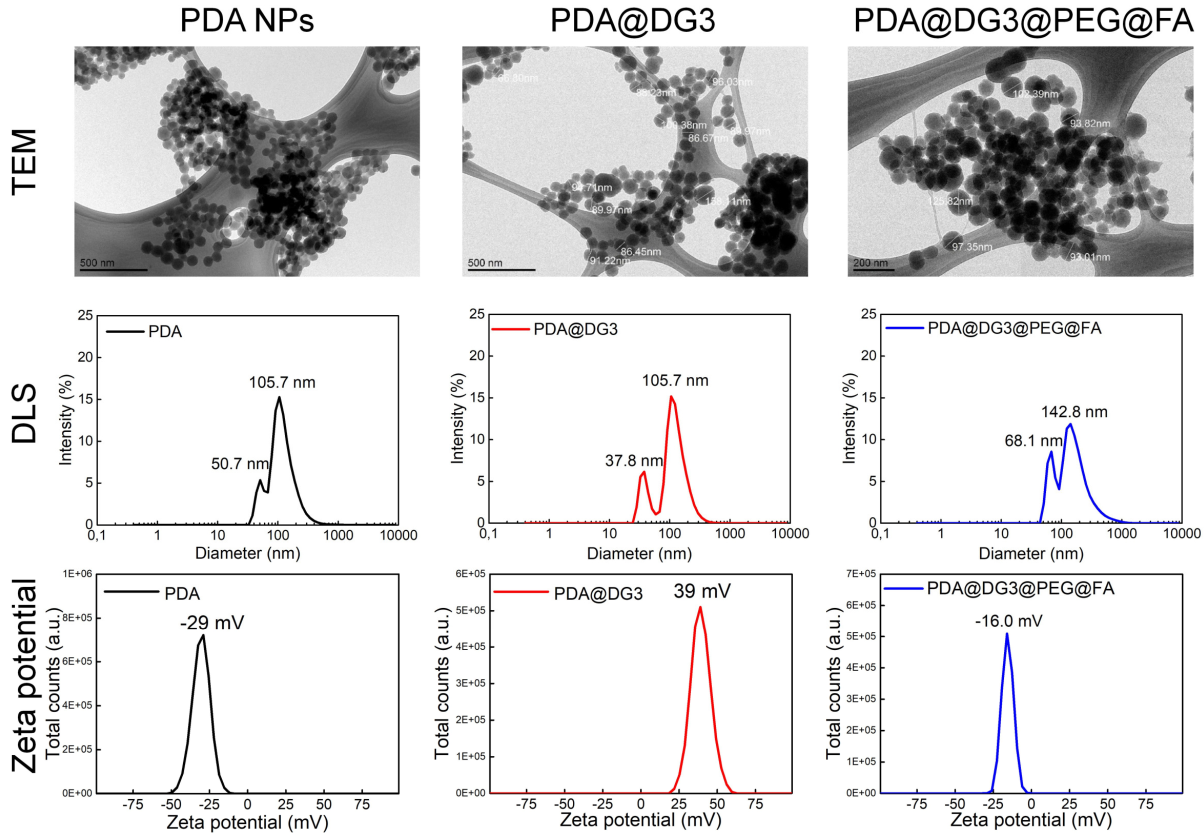

Figure S2. Transmission electron microscopy images, dynamic light scattering and zeta potential measurements of PDA, PDA@DG3 and PDA@DG3@PEG@FA NPs, respectively.

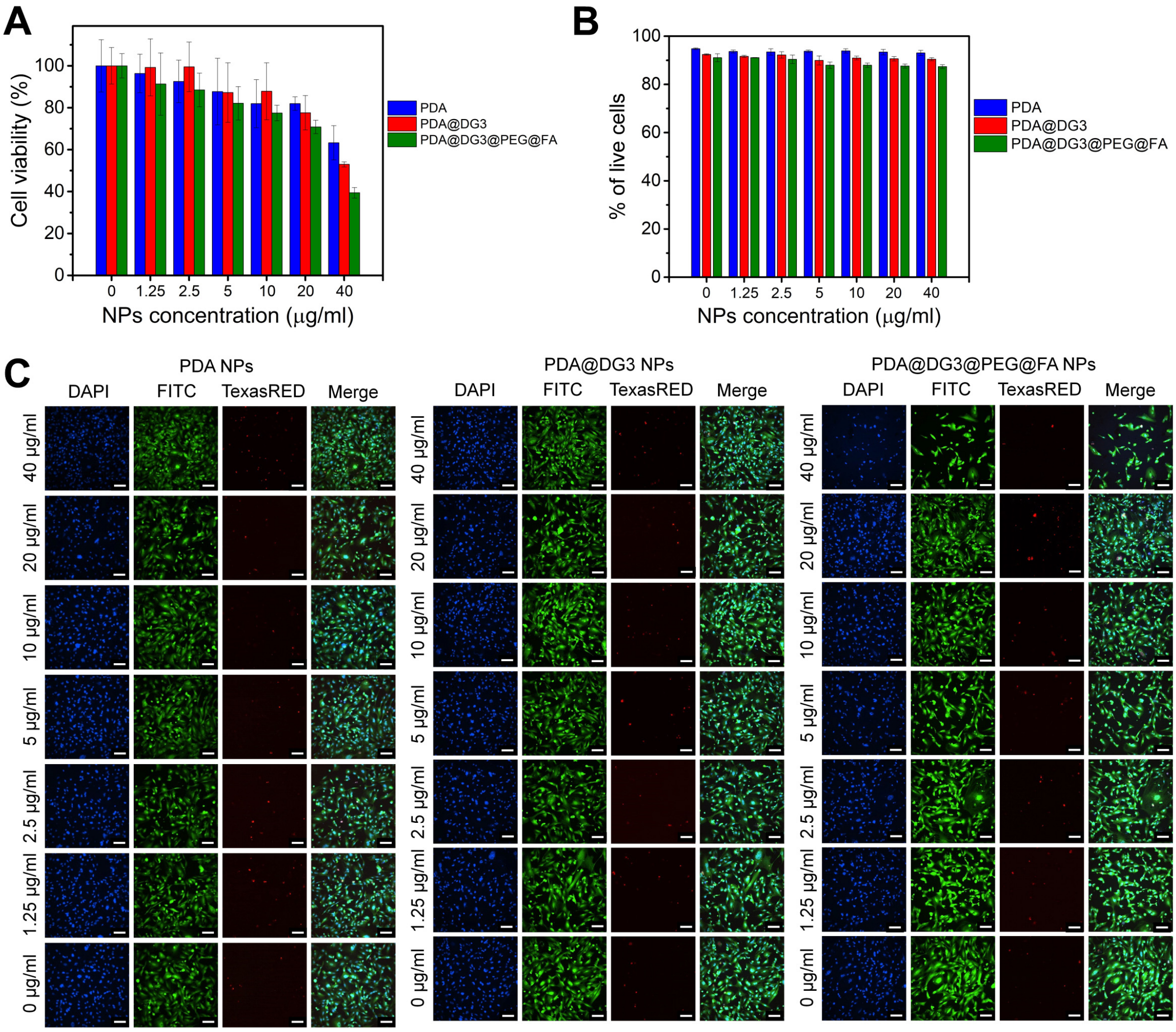

Figure S3. Cell viability assays results of THLE-2 cells incubated for 48 h with PDA, PDA@DG3 and PDA@DG3@PEG@FA NPs. (A) WST-1 cell viability assay results. (B) Live/Dead cell viability assay results. (C) Representative high-content images of THLE-2 cells. The images were obtained using different filters to detect the nuclei (DAPI), live cells (FITC), and dead cells (TexasRed). The scale bars denote 100 µm.

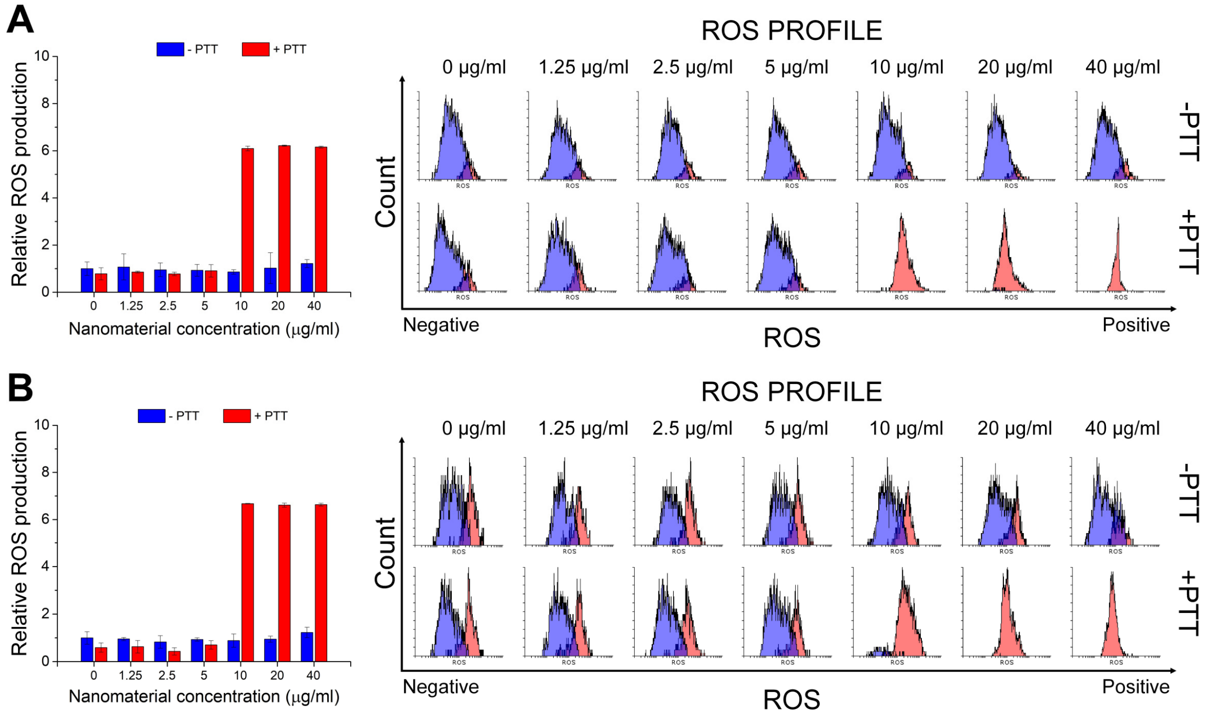

Figure S4. Relative ROS production results and ROS profiles evaluated by flow cytometry for HepG2 cells incubated with PDA@DG3@PEG@FA NPs after 24 h (A) and 48 h (B) of irradiation with 808 nm laser (2 W/cm^2 , 5 min).

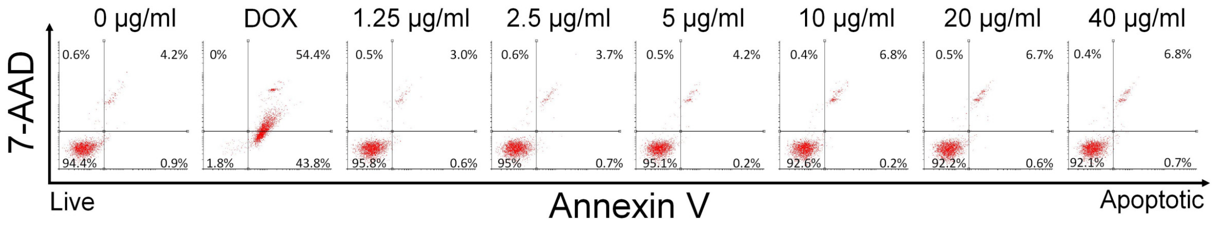

Figure S5. Apoptosis profile of HepG2 cells incubated with PDA@DG3@PEG@FA NPs for 48 h evaluated by flow cytometry.
